# Nrf2 regulates mass accrual and the antioxidant endogenous response in bone differently depending on the sex and age

**DOI:** 10.1371/journal.pone.0171161

**Published:** 2017-02-02

**Authors:** Gretel Gisela Pellegrini, Meloney Cregor, Kevin McAndrews, Cynthya Carolina Morales, Linda Doyle McCabe, George P. McCabe, Munro Peacock, David Burr, Connie Weaver, Teresita Bellido

**Affiliations:** 1 Department of Anatomy and Cell Biology, School of Medicine, Indiana University Purdue University Indianapolis, Indianapolis, Indiana, United States of America; 2 Department of Statistics, Purdue University, West Lafayette, Indiana, United States of America; 3 Department of Medicine, Division of Endocrinology, School of Medicine, Indiana University Purdue University Indianapolis, Indianapolis, Indiana, United States of America; 4 Department of Nutrition Science, Purdue University, West Lafayette, Indiana, United States of America; 5 Roudebush Veterans Administration Medical Center, Indianapolis, Indiana, United States of America; INSERM, FRANCE

## Abstract

Accumulation of reactive oxygen species (ROS) is an important pathogenic mechanism underling the loss of bone mass and strength with aging and other conditions leading to osteoporosis. The transcription factor erythroid 2-related factor2 (Nrf2) plays a central role in activating the cellular response to ROS. Here, we examined the endogenous response of bone regulated by Nrf2, and its relationship with bone mass and architecture in the male and female murine skeleton. Young (3 month-old) and old (15 month-old) Nrf2 knockout (KO) mice of either sex exhibited the expected reduction in Nrf2 mRNA expression compared to wild type (WT) littermates. Nrf2 deletion did not lead to compensatory increase in Nrf1 or Nrf3, other members of this transcription factor family; and instead, Nrf1 expression was lower in KO mice. Compared to the respective WT littermate controls, female KO mice, young and old, exhibited lower expression of both detoxifying and antioxidant enzymes; young male KO mice, displayed lower expression of detoxifying enzymes but not antioxidant enzymes; and old male KO mice showed no differences in either detoxifying or antioxidant enzymes. Moreover, old male WT mice exhibited lower Nrf2 levels, and consequently lower expression of both detoxifying and antioxidant enzymes, compared to old female WT mice. These endogenous antioxidant responses lead to delayed rate of bone acquisition in female KO mice and higher bone acquisition in male KO mice as quantified by DXA and μCT, demonstrating that Nrf2 is required for full bone accrual in the female skeleton but unnecessary and even detrimental in the male skeleton. Therefore, Nrf2 regulates the antioxidant endogenous response and bone accrual differently depending on sex and age. These findings suggest that therapeutic interventions that target Nrf2 could be developed to enhance the endogenous antioxidant response in a sex- and age-selective manner.

## Introduction

Oxidative stress results from the imbalance between free radical generation and the scavenging activity of intracellular antioxidant mechanisms. Accumulation of reactive oxygen species (ROS) causing oxidative damage in different tissues occurs with aging, obesity, menopause, and arthritis, and it is also a crucial pathogenic factor in osteoporosis and metabolic bone diseases [1, 2]. Accumulation of ROS increases osteoclast differentiation directly by activating the transcription factor nuclear factor of activated T–cells, cytoplasmic 1 (NFATc1) in pre-osteoclasts and indirectly by enhancing the expression in cells of the osteoblastic lineage of the receptor activator of nuclear factor kappa-B ligand (RANKL) and tumor necrosis factor α (TNFα), which in turn stimulate osteoclastogenesis [3–5]. Conversely, ROS accumulation decreases the number of osteoblasts by inhibiting their proliferation and differentiation and by inducing premature osteoblast apoptosis [6–9]. In addition, ROS induces apoptosis of osteocytes, the most abundant cells in bone that regulate osteoclast and osteoblast function [10, 11].

The transcription factor erythroid 2-related factor2 (Nrf2) belongs to the Cap-N-Collar family of regulatory proteins that also comprises Nrf1, Nrf3 and p45NFE2 [12]. Nrf2 is ubiquitously expressed and largely responsible for basal and inducible expression of proteins involved in drug metabolism and the cellular response to oxidative stress [13]. The levels of Nrf2 are controlled by ubiquitination and proteosomal degradation. Nrf2 protein is maintained at low levels by its inhibitor, kelch like ECH associated protein 1 (Keap1), which sequesters Nrf2 in the cytosol and facilitates its degradation via the proteasome [13]. Nrf2 is redox-sensitive and under excessive oxidative stress Nrf2 degradation is hindered leading to its accumulation in the cytoplasm and its nuclear translocation [13]. Nrf2 then, binds to antioxidant response elements (AREs) and increases the transcription of cytoprotective genes, which in turn attempt to decrease ROS. Nrf2 regulates the expression of phase II detoxifying enzymes, including NAD(P)H quinone dehydrogenase 1 (NQO1), heme oxygenase 1 (OH-1), ferritin light chain 1 (FTL1) and glutathione S-transferase phosphate 1 (GSTP), which are responsible for indirectly neutralizing ROS by conjugating xenobiotics, increasing their solubility and facilitating their excretion. Nrf2 also controls the expression of antioxidant enzymes, including thio-redoxin reductase 1 (TXNRD1) and superoxide dismutase 1 (SOD1), which directly degrade ROS [14, 15].

Mice in which Nrf2 is globally deleted are viable and exhibit no obvious phenotypic abnormalities [16]. However, consistent with the cytoprotective role of this transcription factor, Nrf2 knockout (KO) mice are more prone to oxidative injury, carcinogenesis and chemical inflammation [17–19], and to develop degenerative diseases associated with elevated oxidative stress including age-related macular degeneration, diabetes, and Parkinson’s disease [20–22].

Earlier studies showed that female Nrf2 KO mice exhibit a deficit in postnatal bone acquisition and increased bone loss [23, 24]. However, controversial results were found when studying male mice, since Park et al reported increased bone mass in Nrf2 KO mice [25], whereas Sun et al reported reduced bone mass and defective anabolic response to bone loading [26]. Therefore, it is still unclear whether Nrf2 regulates bone acquisition and maintenance and if Nrf2 effects are different in the female and male mice skeleton. Moreover, none of the previous studies examined whether the different effects at the bone tissue level are due to a distict endogenous antioxidant response dependent on the sex.

To address these unresolved issues, we examined the bone phenotype of female and male, young and old, Nrf2 KO mice and wild type (WT) littermate controls. Our findings demonstrate that Nrf2 regulates bone accrual differently depending on the sex, being required in the female skeleton but dispensable (and even detrimental) in the male skeleton. Further, the absence of Nrf2 protects from bone loss in old male mice suggesting that Nrf2 negatively influences bone maintenance in the male skeleton. Moreover, the sex-dependent effects of Nrf2 deletion at the tissue level correlate with different antioxidant endogenous responses, as the expression of phase II detoxifying enzymes is strictly dependent on Nrf2 in females but not in males, thus suggesting sex-specific mechanisms for controlling the defense against ROS in bone.

## Materials and methods

### Mice

**Fig 1:**
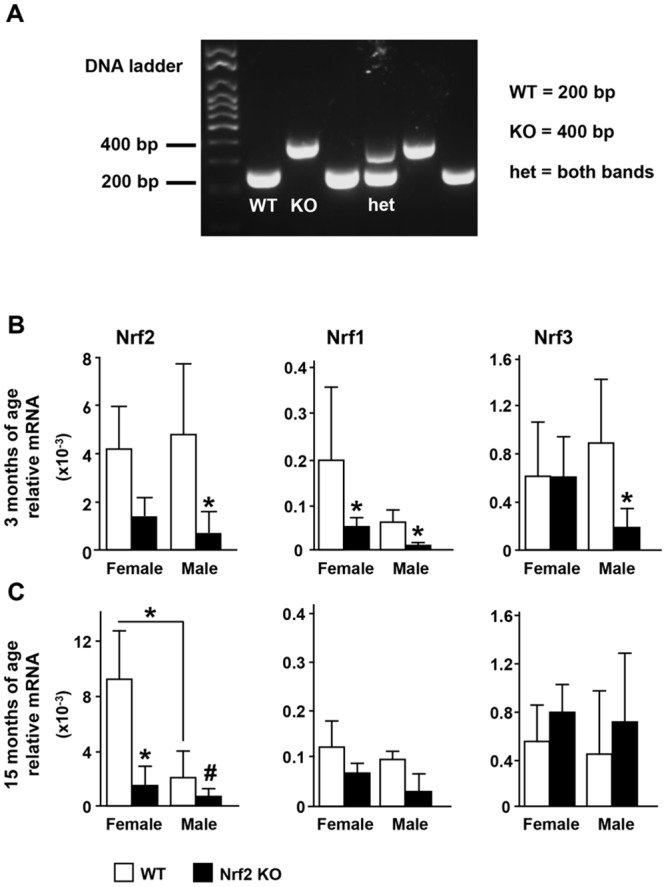
Deletion of the transcription factor Nrf2 does not lead to compensatory increases in Nrf2 or Nrf3. (A) Mice genotyping. PCR products of 200 and 400 bp correspond to WT and KO alleles, respectively. (B, C) Nrf2, Nrf1 and Nrf3 mRNA expression in lumbar vertebra 6 (L6) was quantified by qPCR and normalized by the housekeeping gene GAPDH. Bars represent means ± SD, n = 3–5 mice/ group. * p<0.05 versus respective WT by two-way ANOVA, followed by pairwise multiple comparisons using Tukey method. # p<0.05 vs respective WT by *t*-Test.

WT and Nrf2 KO littermate mice were generated by crossing Nrf2 heterozygous mice (B6.129X1- Nfe2/2^tm1Ywk^/J) purchased from the Jackson Labs. Nrf2 KO mice were originally produced by injection of targeted 129X1/SvJ-derived JM-1 embryonic stem cells into blastocysts, followed by breeding of chimeric mice with C57BL/6 mice [27]; and have been backcrossed more than 10 generations into C57BL/6 strain. Mice were genotyped by PCR of genomic DNA using the following primers: Nrf2 common (GCC TGA GAG CTG TAG GCC C), Nrf2 WT REV (GGA ATG GAA AAT AGC TCC TGC C) and Nrf2 mutant REV (GAC AGT ATC GGC CTC AGG AA), (Invitrogen,Grand Island, NY). Bands were detected by gel electrophoresis corresponding to PCR products of 200 and 400 bp for the WT and KO alleles, respectively (Fig 1A). Mice were housed in the Indiana University Laboratory Animal Research Center (LARC), fed with regular diet (Harlan/ Teklad 7001, Indianapolis, In, USA), received water ad libitum, and maintained on a 12-h light/dark cycle. All animal procedures were approved by the Institutional Animal Care and Use Committee of Indiana University School of Medicine.

### Analysis of the skeletal phenotype

A longitudinal analysis of Nrf2 KO skeletal phenotype was performed in mice until 15 months of age. Bone mineral density (BMD) measurement and micro-CT (μCT) analysis were done as previously described [28, 29]. Mice were anesthetized via inhalation of 2.5% (vol/vol) isoflurane (Abbott Laboratories) mixed with O2 (1.5 L/min), and BMD of the total body, excluding the head and the tail, the lumbar spine (L1–6), and the femur was measured by dual energy X-ray absorptiometry (DXA) by using a PIXImus II densitometer (GE Medical Systems). For μCT analysis, lumbar vertebrae (L5) from 3 and 15-month-old mice were dissected, cleaned of soft tissue, wrapped in gauze with saline, and frozen at -20°C until analyzed at 6 μm resolution using a Scanco μCT 35 instrument (Scanco Medical, Brüttisellen, Switzerland) [30].

### Biochemical markers

Plasma and serum was obtained from blood from the facial vein of mice after 3-h fasting at 2, 5 and 15 months of age. Circulating levels of procollagen type 1 amino-terminal propeptide (P1NP) and C-telopeptide fragments of Type I collagen (CTX) were measured in serum and plasma, respectively, by using ELISA kits (Immunodiagnostic Systems Ltd) [31–33]. Alkaline phosphatase (ALP) was measured in serum on a Randox Daytona analyzer (Randox Laboratories Limited, Northern Ireland, U.K) at the General Clinical Research Center of Indiana University School of Medicine.

### Bone histomorphometry

Vertebrae (L1–L3) from 15 month-old mice were dissected, fixed in 10% buffered formalin, and embedded in methyl methacrylate as published [34]. For dynamic bone histomorphometric analysis, mice were injected IP with calcein (30 mg/kg; Sigma Chemical Co., St. Louis, MO, USA) and alizarin (50mg/kg; Sigma) 12 and 5 days before euthanasia, respectively, as previously described [35]. Thick (100 μm) cross-sections of L1-3 were prepared using a diamond embedded wire saw (Histosaw, Delaware Diamond Knives, Wilmington, DE) and ground to a final thickness of approximately 40 μm. Dynamic histomorphometric analysis was performed on unstained sections, avoiding the primary spongiosa. Total, single and double-labeled perimeter, and inter-label width were measured on the L1-3 vertebrae surfaces. A value of 0.1 μm/d was used for mineral apposition rate (MAR) when single labels were present but double labels were not detected [36]. Static bone histomorphometric analysis was performed in longitudinal sections of L1-3 vertebrae stained for von Kossa and counterstained with toluidine blue. All measurements were obtained using a semiautomatic analysis system, OsteoMeasure high-resolution digital video system (OsteoMetrics Inc., Decatur, GA) attached to a microscope equipped with an ultraviolet light source (Nikon Optiphot 2 microscope, Melville, NY). The terminology and units used are those recommended by the Histomorphometry Nomenclature Committee of the American Society for Bone and Mineral Research [37].

### Quantitative PCR

Total RNA extraction and quantitative PCR (qPCR) were performed as reported previously [29]. Briefly, total RNA was extracted from lumbar vertebrae L6 by using TRIzol (Invitrogen,Grand Island, NY) and reverse-transcribed the RNA using the High-Capacity cDNA Archive Kit (Applied Biosystems, Foster City, CA) according to the manufacturer’s instructions. Gene expression was analyzed by qPCR using primer probe sets from Applied Biosystems or from Roche Applied Science (Roche Applied Science, Indianapolis, IN). Relative mRNA expression levels were normalized to the house-keeping gene GAPDH by using the ΔCt method [28, 29].

### Statistical analysis

Gene expression, μCT, bone markers and histological measurements were reported as the mean ± standard deviation. Differences were evaluated by 2x2 two-way ANOVA with factors sex and genotype. If a significant main effect or interaction was found, the Tukey-Kramer multiple comparison procedure was used to examine pairwise differences among the four treatment combinations. Student’s t test was used to analyze differences between WT and Nrf2 KO for each sex. P values <0.05 were considered statistically significant.

Longitudinal profiles of total, femoral and spinal BMD measurements were estimated as mean ± standard error. For each gender by genotype combination, the longitudinal profiles of total, femoral, and spinal BMD were modeled as a nonlinear mixed model with four parameters, b0, b1, b2, and b3 that varied from mouse to mouse. This model is a spline that increases with age up to b2, the point of peak bone mass, and then levels out or decreases. The first part of the spline is of the form b0+b1*(1/age) and describes the relationship for age less than or equal to b2. The second part is of the form b0+(b1/b2)+b3*(age-b2) and describes the relationship for age greater than b2. In this model, b0+b1 corresponds to BMD at 1 month, b1 represents the curvature of the relationship from 1 month up to b2, and b3 is the slope of the relationship for age greater than b2. Note that b1 is negative because BMD increases as 1/age decreases. Data for all mice were included in a single model that allowed each of the four parameters to vary according to the design. We performed two-way significance tests (main effects of sex and genotype and the sex by genotype interaction) for each parameter. Based on the results of these significance tests, final models were run with selected parameters constrained to be equal in subsets of the four design groups. Using these models, estimates and standard errors of BMD at age 1 month (b0+b1), curvature (b1), age at peak bone mass (b2), slope after peak (b3), BMD at peak ((b0+(b1/b2)), and BMD at age 15 months ((b0+(b1/b2)+b3*(15-b2)) were calculated. Analyses were performed using SigmaStat (SPSS Science) and PROC NLMIXED in SAS 9.3 (SAS Institute, Cary, NC).

## Results

### The absence of Nrf2 does not induce a compensatory effect of Nrf1 and Nrf3 gene expression

Mice were genotyped as detailed in methods section (Fig 1A). Young (3 month-old) and old (15 month-old) KO mice of either sex exhibited the expected reduction in Nrf2 mRNA expression compared with WT littermates, as demonstrated by mRNA expression in bone (Fig 1B and 1C). In addition, old WT male mice exhibited lower Nrf2 expression than old female WT mice (Fig 1B and 1C).

We next investigated whether Nrf2 deletion lead to compensatory effects by increasing the expression of Nrf1 or Nrf3. The lack of Nrf2 decreased Nrf1 mRNA expression in female and male KO bones, although changes showed statistical significance only in young mice (Fig 1A and 1B). Further, Nrf2 deletion did not alter Nrf3 expression in female bone or in old male bone, but decreased it in bones from young male Nrf2 KO mice. Therefore, Nrf2 deletion does not lead to compensatory increases in Nrf1 or Nrf3; but instead Nrf2 deletion leads to decreases in Nrf1 and Nrf3 expression depending on the sex and age.

### Cytoprotective proteins genes are differentially regulated by Nrf2 deletion in the female and male skeleton

**Fig 2:**
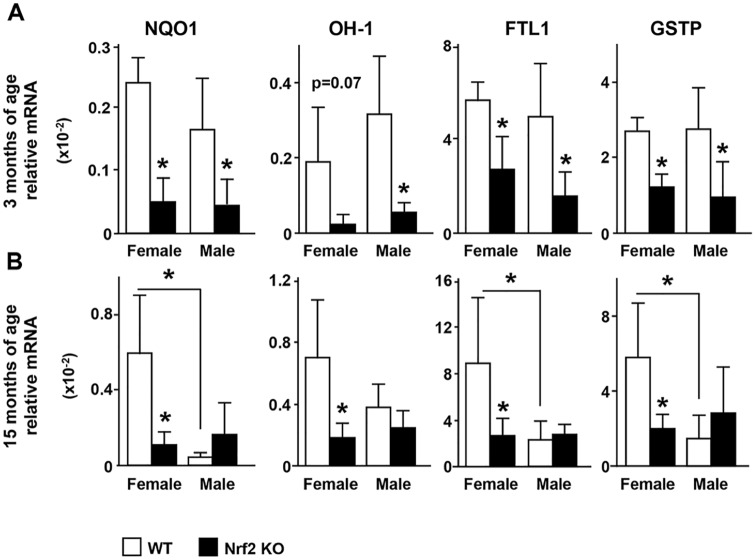
Phase II detoxifying enzyme expression in bone depends on Nrf2 in female and male mice. The mRNA expression of phase II detoxifying enzymes was quantified by qPCR and normalized by GAPDH in L6 of 3 month-old (A) and 15 month-old (B) mice. Bars represent means ± SD, n = 3–5 mice/ group. * p<0.05 versus respective WT by two-way ANOVA, followed by pairwise multiple comparisons using Tukey method.

It is known that in other tissues Nrf2 regulates phase II detoxifying enzymes and antioxidant enzymes. We found that indeed the expression of phase II detoxifying enzymes in bone also depends on Nrf2. Thus, transcripts for NQO1, OH-1, FTL1 and GSTP, were downregulated in young and old female KO mice and young male KO mice (Fig 2A and 2B). In addition, similar to the lower Nrf2 expression exhibited by WT old male mice (Fig 1C), WT old male mice exhibited lower expression of phase II detoxifying enzymes compared with old WT female mice (Fig 2B). Moreover, the levels of these mRNA transcripts were not further decreased by Nrf2 deletion in old male mice.

**Fig 3:**
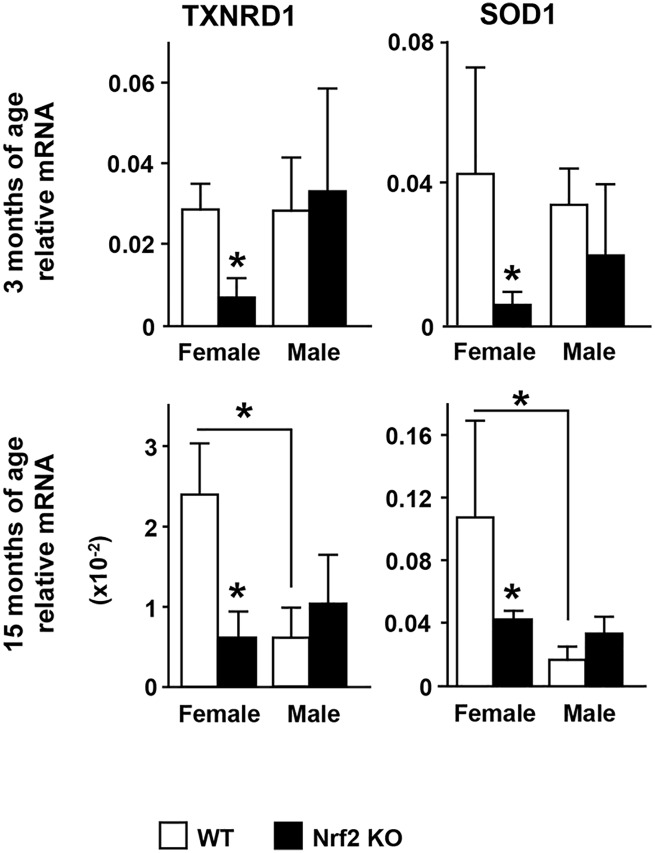
The expression of the antioxidant enzymes is dependent on Nrf2 in female but not in male mice. Expression of antioxidant enzymes was quantified as in Fig 2. Bars represent means ± SD, n = 3–5 mice/ group. * p<0.05 versus respective WT by two-way ANOVA, followed by pairwise multiple comparisons using Tukey method.

Similar to the regulation of phase II detoxifying enzymes, the expression of the antioxidant enzymes TXNRD1 and SOD1 was lower in female KO mice of either age (Fig 3). In contrast, TXNRD1 and SOD1 expression was not different in male KO bone of either age compared to the respective littermate WT. However, the expression of these antioxidant enzymes was lower in old WT male mice compared with old WT female mice, resembling the pattern followed by the expression of phase II detoxifying enzymes and Nrf2 itself. These results demonstrate that phase II detoxifying and antioxidant enzymes are dependent on Nrf2 in female mice, whereas in male mice, the expression of antioxidant enzymes appears to be controlled by alternative mechanisms.

### The transcription factor Nrf2 is required for bone acquisition in a sex-specific manner

**Fig 4:**
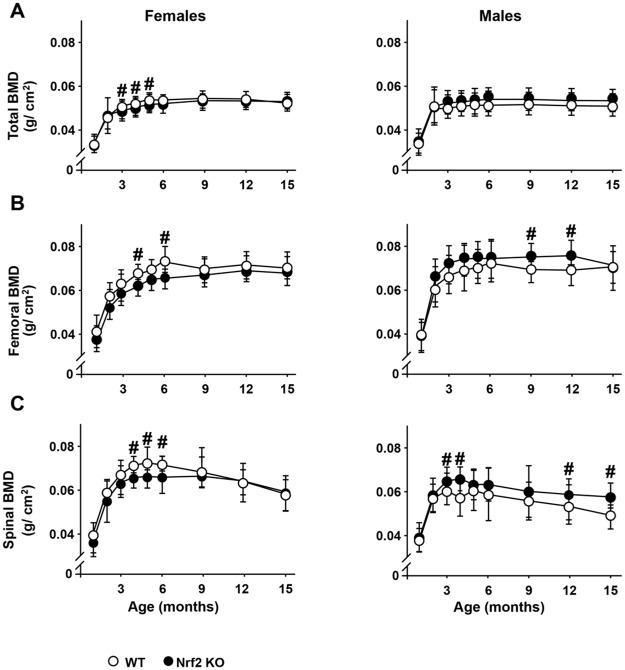
Nrf2 regulates bone mass by sex specific mechanisms. Longitudinal analysis of total (A), femoral (B) and spinal (C) BMD up to 15 month of age in Nrf2 KO and WT littermate female and male mice. BMD was assessed monthly by DEXA, n = 9–15 mice/ group. # p<0.05 versus respective WT by *t*- Test.

To determine the consequences of Nrf2 deletion on bone accrual and maintenance, we performed a longitudinal analysis of total, femoral and spinal BMD in cohorts of WT and Nrf2 KO male and female mice (Fig 4). Female KO mice exhibited lower BMD (total, femoral and spinal) at 3–6 month of age compared with littermate WT female mice. In contrast, no differences were detected in total BMD at any age in male KO mice. However, they exhibited higher spinal BMD at 3 and 5 months of age, which remained elevated at 12 and 15 months of age. Higher femoral BMD at 9 and 12 months of age were also observed in male KO mice compared with the respective WT littermates.

**Fig 5:**
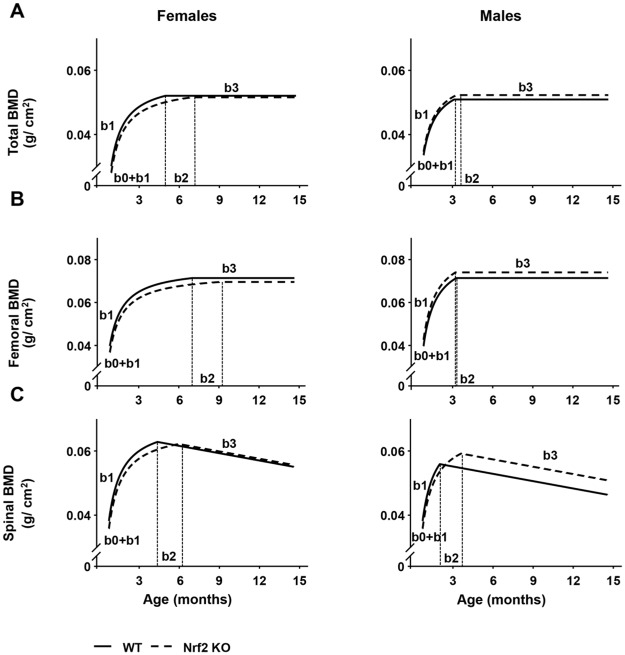
Nrf2 is required for bone acquisition in a sex-specific manner. Longitudinal profiles of total, femoral and spinal BMD measurements were estimated as mean ± standard error, n = 9–15 mice/ group. Estimates and standard errors of BMD at age 1 month (b0+b1), curvature (b1), age at peak bone mass (b2), slope after peak (b3), BMD at peak ((b0+(b1/b2)), and BMD at age 15 months ((b0+(b1/b2)+b3*(15-b2)) were calculated. For each sex by genotype combination the longitudinal profiles of total, femoral, and spinal BMD were modeled as a nonlinear mixed model with four parameters, b0, b1, b2, and b3 that varied from mouse to mouse. The first part of the spline is of the form b0+b1*(1/age) and describes the relationship for age less than or equal to b2. The second part is of the form b0+(b1/b2)+b3*(age-b2) and describes the relationship for age greater than b2.

BMD values were modeled using a nonlinear mixed model (Fig 5). KO female mice exhibited lower initial BMD (at 1 month of age) than WT littermates, whereas no differences were detected in male mice at this age (Table 1A and Fig 5). The early rate of total bone accrual (b1) did not vary with sex or genotype. Although female KO mice reached adult peak of bone mass (b2) 1.98 months later than female WT mice, they both attained the same peak of bone mass (Fig 5A). On the other hand, male KO mice attained 2.63% higher peak of total bone mass than WT littermates, while the age at peak of bone mass did not differ. In this model, the parameter b3 was set to zero indicating that there was no change in BMD from peak to 15 months.

**Table 1:** Longitudinal analysis of BMD in KO mice and WT littermates. In this model, b0+b1 corresponds to BMD at age = 1 month, b1 represents the curvature of the relationship from 1 month up to b2, b2 is the age at peak bone mass, and b3 is the slope of the relationship for age greater than b2. Two-way significance tests (main effects of gender and genotype and the gender by genotype interaction) for each parameter were performed. Estimates and standard errors of BMD at age 1 month (b0+b1), curvature (b1), age at peak bone mass (b2), slope after peak (b3), BMD at peak ((b0+(b1/b2)), and BMD at age 15 months ((b0+(b1/b2)+b3*(15-b2)) were calculated.

**A Total**	**Females**		**Males**	
	**WT**	**Nrf2 KO**	**WT**	**Nrf2 KO**
**BMD at 1 month (b0+b1)**	0.0338 ± 0.0004	0.0320 ± 0.0005 a	0.0338 ± 0.0004	0.0348 ± 0.0005
**Curvature (b1)**	-0.0246 ± 0.0006	-0.0246 ± 0.0006	-0.0246 ± 0.0006	-0.0246 ± 0.0006
**Age at peak bone mass (b2)**	5.1071 ± 0.4828	7.0971 ± 0.6366 b	3.3311 ± 0.2169	3.5002 ± 0.2594
**Slope after peak (b3)**	0	0	0	0
**BMD at peak (b0+(b1/b2)**	0.0536 ± 0.0003	0.0532 ± 0.0002	0.0510 ± 0.0002	0.0524 ± 0.0001 a
BMD at 15 months (b0+(b1/b2))+b3*(15-b2))	0.0536 ± 0.0003	0.0532 ± 0.0002	0.0519 ± 0.0002	0.0463 ± 0.0001 a
**B Femur**	**Females**		**Males**	
	**WT**	**Nrf2 KO**	**WT**	**Nrf2 KO**
**BMD at 1 month (b0+b1)**	0.0397 ± 0.0115	0.0368 ± 0.0011 a	0.0397 ± 0.0013	0.0426 ± 0.0013 a
**Curvature (b1)**	-0.0366 ± 0.0006	-0.0366 ± 0.0006	-0.0443 ± 0.0022	-0.0443 ± 0.0022
**Age at peak bone mass (b2)**	7.2510 ± 0.1502	9.3564 ± 0.1533 b	3.4760 ± 0.2989	3.4184 ± 0.3052
**Slope after peak (b3)**	0	0	0	0
**BMD at peak (b0+(b1/b2)**	0.0713 ± 0.0005	0.0695 ± 0.0005 a	0.0714 ± 0.0003	0.0740 ± 0.0005 a
BMD at 15 months (b0+(b1/b2))+b3*(15-b2))	0.0713 ± 0.0005	0.0695 ± 0.0005 a	0.0714± 0.0003	0.0740 ± 0.0005 a
**C Spine**	**Females**		**Males**	
	**WT**	**Nrf2 KO**	**WT**	**Nrf2 KO**
**BMD at 1 month (b0+b1)**	0.3823 ± 0.0009	0.0358 ± 0.0010 a	0.3823 ± 0.0009	0.0358 ± 0.0010 a
**Curvature (b1)**	-0.0312 ± 0.0015	-0.0312 ± 0.0015 b	-0.0312 ± 0.0015	-0.0312 ± 0.0015 b
**Age at peak bone mass (b2)**	4.6757 ± 0.4935	6.2914 ± 0.5845	2.3018 ± 0.1504	3.9176 ± 0.3261
**Slope after peak (b3)**	0.0007 ± 0.00008	0.0007 ± 0.00008	0.0007 ± 0.00008	0.0007 ± 0.00008
**BMD at peak (b0+(b1/b2))**	0.0628 ± 0.0006	0.0621 ± 0.0005	0.0559 ± 0.0006	0.0576 ± 0.0006 b
BMD at 15 months (b0+(b1/b2))+b3*(15-b2))	0.0550 ± 0.0008	0.0544 ± 0.0007	0.0481 ± 0.0006	0.0499 ± 0.0008 b

^a^ p<0.05 vs respective WT

^b^ p<0.001 vs respective WT

Similarly to total BMD, female KO mice showed lower femoral BMD at 1 month of age compared with WT mice, but male KO mice displayed higher BMD than their WT littermates (Table 1B and Fig 5B). Female KO mice attained peak femoral bone mass 2.10 months later than WT littermates, whereas males from both genotypes attained peak bone mass at the same age. Female KO mice attained 2.53% lower BMD in the femur than female WT mice at peak of bone mass. Once they reached peak bone mass in the femur, females and males from each genotype maintained it until the end of the experiment at 15 months of age.

Longitudinal analysis of BMD in the spine showed lower initial BMD (b0+b1) in KO mice from both sexes, while the early rate of bone accrual (b1) did not vary with sex or genotype (Table 1C and Fig 5C). Female and male KO mice reached adult peak spinal bone mass (b2) 1.61 months later than WT mice of the same sex (Fig 5A, 5B and 5C). No differences were found in peak spinal bone mass in female mice, whereas male KO mice attained a 3.1% higher peak than WT littermates (Fig 5C). After reaching peak bone mass, spinal BMD decreased in all mice, at a common slope of 0.00075 g/cm^2^/month (b3) (Table 1C). Thus, female WT mice lost 12.32% and female KO mice lost 12.45% of spinal BMD while male WT mice lost 13.84% and KO mice lost 13.42% (Fig 5C).

**Fig 6:**
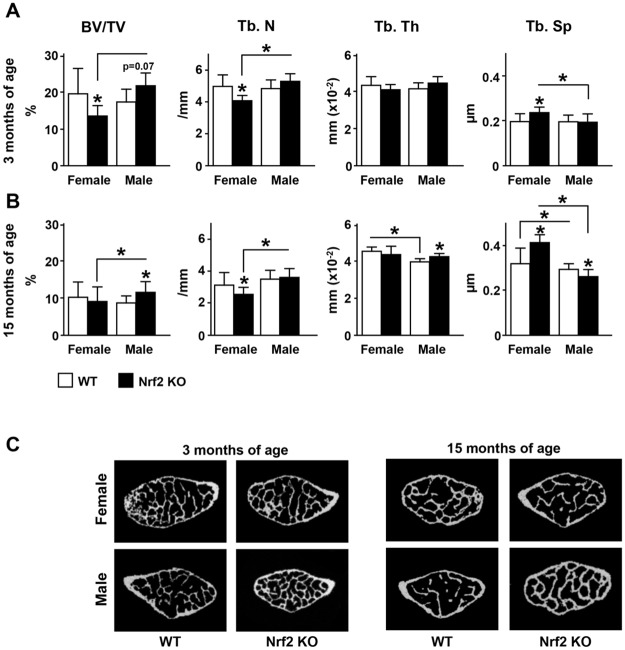
Nrf2 is requiered for full bone accrual in the female skeleton but unnecessary, and even detrimental, in the male skeleton. μCT analysis of cancellous bone microarchitecture in L5 vertebrae in 3-month-old mice (A) and in 15-month-old mice (B). Bars represent means ± SD, n = 7–8 or 11–15 mice/ group, respectively. * p<0.05 versus respective WT by two-way ANOVA, followed by pairwise multiple comparisons using Tukey method. Representative images of vertebrae from young and old female and male Nrf2 KO and wild type littermate controls are shown in (C).

Consistent with the BMD results, μCT analysis of lumbar vertebra (L5) showed that Nrf2 deficiency affected bone micro-architecture. Thus, young female KO mice exhibited lower bone volume (BV/TV) compared with WT littermates (Fig 6A and 6B, and Table 2). In addition, both young and old female KO exhibited lower trabecular number (Tb. N) and higher trabecular spacing (Tb. Sp). In contrast, old male KO mice exhibited higher bone volume and trabecular thickness (Tb. Th) and lower Tb. Sp compared with WT littermates. Taken together, these findings demonstrate an opposite effect of Nrf2 on bone acquisition depending on the sex.

**Table 2:** Micro-CT analysis of Nrf2 KO mice and WT littermates. Total volume (TV), bone volume (BV), connectivity density (CONN d), structure model index (SMI) of lumbar vertebrae (L5) was measured in cancellous bone of young (3 month-old) and old (15 month-old) female and male WT of Nrf2 KO mice. The numbers represent means ± SD, n = 6–9 or 9–12 mice/ group for young and old mice, respectively.

	3 month-old				15 month-old			
	WT		Nrf2 KO		WT		Nrf2 KO	
	Female	Male	Female	Male	Female	Male	Female	Male
**TV (mm**^**3**^**)**	0.98±0.08	1.01±0.05	1.03±0.64	1.04±0.10	1.09±0.09	1.16±0.09	1.11±0.16	1.12±0.14
**BV (mm**^**3**^**)**	0.18±0.06	0.17±0.03	0.14±0.03 a	0.22±0.05 a, b	0.12±0.05	0.10±0.02	0.12±0.08	0.12±0.04
**CONN d (1/(mm**^**3**^**)**	169.20±56.60	187.70±37.50	137.90±35.00	216.65±37.90 b	32.00±16.81	72.07±16.80 b	43.00±18.24	64.82±14.00 b
**SMI**	1.32±0.50	1.40±0.35	1.77±0.40	1.36±0.50	1.85±0.44	2.06±0.44	1.86±0.48	2.00±0.30
**Mean/Density of BV (material) [mg HA/ccm]**	829.90±24.30	848.40±14.20	827.34±22.80	832.90±22.15	876.22±27.33	862.46±23.15	877.53±22.70	867.67±19.22

^a^ p<0.05 versus respective WT and

^b^ p<0.05 versus female mice, by two-way ANOVA, followed by pairwise multiple comparisons using Tukey method.

### Nrf2 deletion increases bone remodeling markers or bone formation markers in female or male Nrf2 KO mice, respectively, without significantly altering tissue bone formation indexes

**Fig 7:**
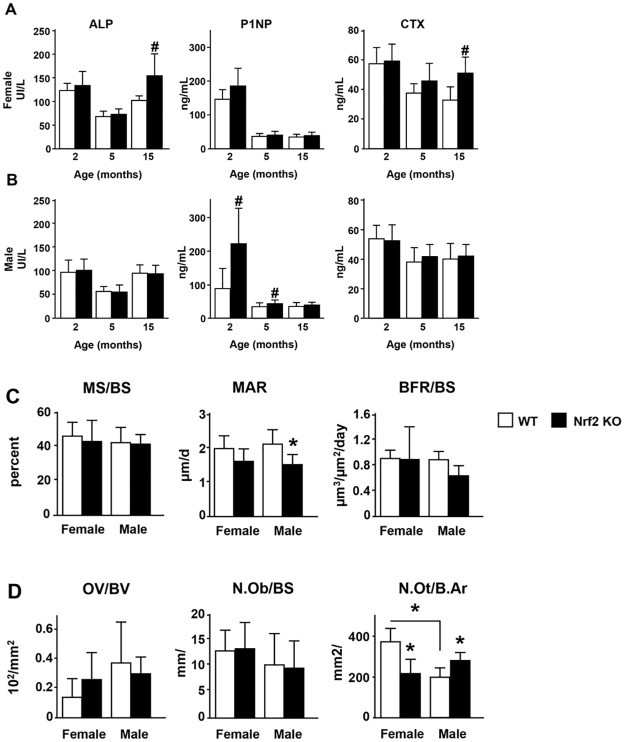
Nrf2 deletion increases bone remodeling markers or bone formation markers in female or male Nrf2 KO mice, respectively, without significantly altering tissue bone formation indexes. Circulating levels of alkaline phosphatase (ALP), procollagen type 1 amino-terminal propeptide (P1NP) and C-telopeptide fragments (CTX) were measured in female (A) and male (B) WT and Nrf2 KO mice at the indicated ages. Bars are means ± S.D, n = 8–10 mice/ group. # p<0.05 by *t-* Test. Dynamic (C) and static (D) histomorphometric parameters were scored in L1-L3 lumbar vertebral bone sections of 15-month-old WT and Nrf2 KO male mice. Mineralizing surface (MS)/BS, mineral apposition rate (MAR), and bone formation rate (BFR)/BS were quantified in unstained sections. Osteoid volume (OV)/BS, number of osteoblasts (N.Ob)/BS, and number of osteocytes (N.Ot)/ B.Ar were measured in sections stained with von Kossa and counterstained with toloudine blue. Bars are means ± S.D, n = 6–8 mice/ group. * p<0.05 versus respective WT by two-way ANOVA, followed by pairwise multiple comparisons using Tukey method.

At 15 months of age, female KO mice exhibited higher circulating levels of the osteoblast marker ALP and the resorption marker CTX, but no changes in P1NP, a bonafide marker of bone formation P1NP, although no changes in any of these markers were detected in younger mice (Fig 7A). Consistent with the higher bone accrual in male KO mice, circulating P1NP was higher at 2 and 5 months of age, although it was not different at 15 months of age compared to WT littermates (Fig 7B). However, no differences between male KO and WT littermates were detected in ALP or CTX at any age. Histomorphometric analysis showed that bone formation indexes, osteoid volume, and osteoblast number were not different in male KO mice at 15 month of age compared with WT littermates, except for a significantly lower MAR in male KO mice (Fig 7C and 7D). Osteocyte density was lower in male compared with female WT mice. In addition, female KO mice exhibited lower, whereas male KO mice exhibited higher osteocyte density compared with the respective WT littermate mice (Fig 7D).

**Fig 8:**
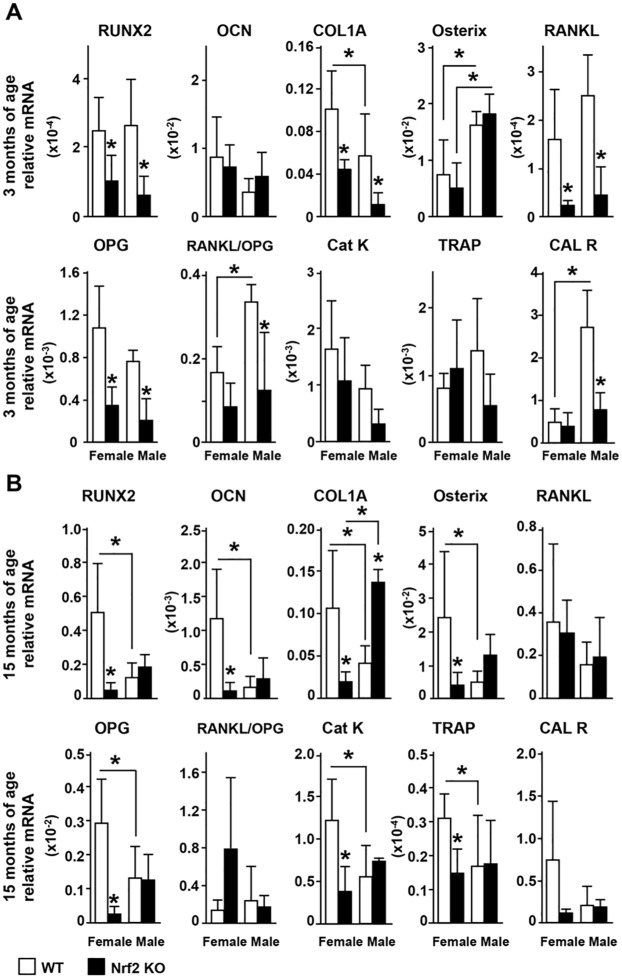
Osteoclast and osteoblast gene expression in Nrf2 KO and WT mice. mRNA expression was measured and expressed as in Figs 1–3. Bars represent means ± SD, n = 3–5 mice/ group. * p<0.05 versus respective WT by two-way ANOVA, followed by pairwise multiple comparisons using Tukey method.

Gene expression analysis showed similarities as well as differences between the expression of osteoblast and osteoclast genes in female versus male KO mice (Fig 8). In female KO mice, Runx2, collagen 1A and OPG expression is reduced in both 3 and 15 month-old mice; whereas RANKL expression is reduced in the young mice and osteocalcin, osterix, cathepsin K and TRAP expression is reduced in the old mice. Young male KO mice exhibited similar decreases in these osteoblast and osteoclast markers compared to female mice, except that calcitonin receptor expression was decreased. Further, old male KO mice exhibited increased expression of collagen 1A but no other changes in gene expression compared to WT littermates of the same age. No differences in the expression of Sost or the Wnt target gene axin 2 were found in bones of mice of either sex at any age (not shown).

## Discussion

**Fig 9:**
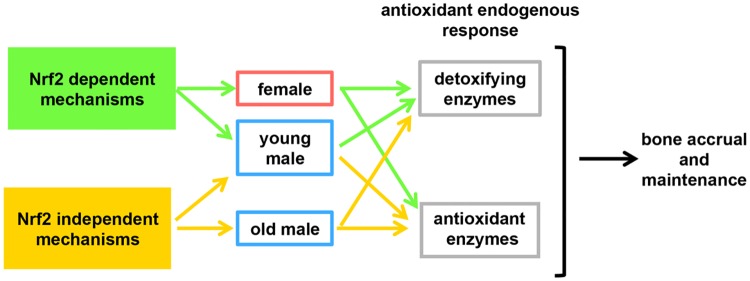
Nrf2 regulates the antioxidant endogenous response and bone accrual differently depending on the sex and age. Scheme summarizing the findings of the study. In female young and old mice, Nrf2 dependent mechanisms are responsible for regulating the expression of detoxifying and antioxidant enzymes in bone. In young male mice, Nrf2 dependent mechanisms regulate the expression of detoxifying enzymes whereas Nrf2 independent mechanisms regulate antioxidant enzymes. Nrf2 independent mechanisms are responsible for the full antioxidant endogenous response, in old male mice, regulating both detoxifying and antioxidant enzymes.

In this study, we report the endogenous antioxidant response in bone and its correlation with the skeletal phenotype of mice with global deletion of Nrf2, a transcription factor with purported role in bone mass homeostasis. Our findings demostrate that genes responsible for cellular protection from the damaging effects of ROS are regulated by Nrf2 depending on the sex and age. We found that in female young and old mice, Nrf2-dependent mechanisms are responsible for regulating the expression of detoxifying and antioxidant enzymes; in young male mice, Nrf2-dependent mechanisms regulate the expression of detoxifying enzymes whereas Nrf2-independent mechanisms regulate antioxidant enzymes; and lastly, in old male mice, Nrf2-independent mechanisms are responsible for the full antioxidant endogenous response, regulating both detoxifying and antioxidant enzymes in bone (Fig 9). The dissimilar regulation of cytoprotective genes correlates with different effects of Nrf2 on bone accrual and maintenance depending on the sex. Thus, there is delayed rate of bone acquisition in female but higher bone acquisition in male KO mice demonstrating the requirement of Nrf2 for full bone accrual in the female skeleton but not in the male skeleton. Our findings suggest that therapeutic interventions that target Nrf2 could be developed to facilitate the defense against the damagind effects of ROS in a sex- and age-selective manner.

The results of our study provide clarification on the conflicting previous reports showing sexual dimorphism in the skeletal phenotype of Nrf2 KO mice [23–26]. Kim et al showed by μCT performed at 3, 6 and 8 weeks of age that female Nrf2 KO mice of C57BL/J6 background exhibit reduced bone mass in distal femur [23]. Ibañez et al reported that female KO mice of the same strain display reduced bone quality compared to WT mice [24]. These results are consistent with our current findings in female KO mice. In contrast to our findings with male mice, Sun et al reported that male Nrf2 KO mice of C57BL6/129SV mixed background exhibited lower BMD than WT littermates [26]. However, Park et al reported that at 9 weeks of age KO mice of the same mixed background display higher femoral bone mass and increased BV/TV by μCT analysis, although the gender of the animals was not specified in his report [25]. The discrepancies among these previous studies could be due to the fact that mice of different sex or age were examined and, in view of our findings, a different antioxidant response in bone would be expected. The mechanistic bases by which Nrf2 modulates the cellular response to ROS differently in females and males are unknown. Potentially, crosstalk between Nrf2 activation and signaling downstream of the sex steroid receptors could contribute to the divergent regulation of cytoprotective genes in female as compared with male bone. Indeed, estrogen-dependent signaling increases Nrf2 activity in several cell types [38–40]. The fact that maintaining an optimal antioxidant response in bone is dependent on Nrf2 expression in females but not in males suggests that mechanisms other than Nrf2 defend against oxidative stress in the male skeleton. Among the mechanisms that counteract the adverse consequences of oxidative stress, the forkhead box O (FoxO) transcription factors have been shown to regulate the expression of antioxidant enzymes and regulate skeletal homeostasis [41]. Sirtuin1 (Sirt1), a nicotinamide adenine dinucleotide oxidized (NAD+)-dependent class III deacetylase, regulates the activity of FoxOs, and changes in the levels of Sirt1 expression are associated with the onset of degenerative diseases characterized by increased ROS, including osteoporosis [42, 43]. Whether these mechanisms confer cellular protection in a sex-dependent manner remains unknown. Future studies are warranted to investigate whether differences in the levels of Sirt1 or in the activity of FoxOs could explain the different response of bone to Nrf2 deficiency between females and males.

The potential implications of the decreased endogenous antioxidant response for the skeletal phenotype in mice lacking Nrf2 remain unknown. However, the fact that female Nrf2 KO mice exhibit deficient bone accrual compaired to WT littermate mice points to an important role in Nrf2-dependent anti-oxidant and detoxifying enzymes in bone acquisition in the female skeleton. Remarkably, however, Nrf2-dependent events appear not to be relevant for bone accrual in the male skeleton pointing to alternative mechanisms controlling ROS balance in this sex. We cannot exclude the possibility that at least part of the differential bone response to the absence of Nrf2 in males versus females could be secondary to systemic effects rather than being due to a bone cell-autonomous response since the Nrf2 KO mice lack Nrf2 expression and function in all tissues. Future studies with tissue and cell specific KO are warranted to distinguish between these two possibilities. In contrast to bone accrual that is dependent on Nrf2 in a sex-dependent manner, bone maintenance appears to be independent of Nrf2 in both sexes, as the rate of bone loss after adult peak bone mass is similar in the female and male skeleton in both WT and KO mice.

Overall, the basal phenotype of KO mice is mild suggesting that under physiological conditions Nrf2-dependent signaling does not have major consequences for bone accrual and maintenance. However, exaggerated ROS production has been shown to be an important factor for the bone loss that ensues with sex steroid deficiency, aging and excess of glucocorticoids [1–3, 44]. This evidence raises the possibility that the differences observed under basal conditions could be exacerbated in pathological conditions in which ROS accumulates. Future studies will be required to directly test this possibility.
